# Haplotype analysis suggest common founders in carriers of the recurrent *BRCA2 *mutation, 3398delAAAAG, in French Canadian hereditary breast and/ovarian cancer families

**DOI:** 10.1186/1471-2350-7-23

**Published:** 2006-03-15

**Authors:** Kathleen K Oros, Guy Leblanc, Suzanna L Arcand, Zhen Shen, Chantal Perret, Anne-Marie Mes-Masson, William D Foulkes, Parviz Ghadirian, Diane Provencher, Patricia N Tonin

**Affiliations:** 1 Department of Human Genetics, McGill University, Montreal, Canada; 2 Program of Surgical oncology, Department of Oncology, McGill University, Montreal, Canada; 3 Research Institute of the McGill University Health Centre, Montreal, Canada; 4 Centre de recherche du Centre Hospitalier de l'Université de Montréal/Institut du cancer de Montréal, Hôpital Notre-Dame, Montréal, Canada; 5 Département de médicine, Université de Montréal, Montreal, Canada; 6 Program in Cancer Genetics, Departments of Oncology and Medicine, McGill University, Montreal, Canada; 7 Unité de recherché en épidémiologie, Centre Hospitalier de l'Université de Montréal, Hôpital Hôtel-Dieu, Faculté de médicine, Université de Montréal, Montréal, Canada; 8 Division de gynécologie oncologique, Université de Montréal, Montreal, Canada

## Abstract

**Background:**

The 3398delAAAAG mutation in *BRCA2 *was recently found to recur in breast and/or ovarian cancer families from the French Canadian population of Quebec, a population that has genetic attributes consistent with a founder effect. To characterize the contribution of this mutation in this population, this study established the frequency of this mutation in breast and ovarian cancer cases unselected for family history of cancer, and determined if mutation carriers shared a common ancestry.

**Methods:**

The frequency was estimated by assaying the mutation in series of French Canadian breast cancer cases diagnosed before age 41 (n = 60) or 80 (n = 127) years of age, and ovarian cancer cases (n = 80) unselected for family history of cancer by mutation analysis. Haplotype analysis was performed to determine if mutation carriers shared a common ancestry. Members from 11 families were analyzed using six polymorphic microsatellite markers (cen-*D13S260*-*D13S1699*-*D13S1698*-*D13S1697*-*D13S1701*-*D13S171*-tel) spanning approximately a 3.6 cM interval at the chromosomal region 13q13.1, which contains *BRCA2*. Allele frequencies were estimated by genotyping 47 unaffected female individuals derived from the same population. Haplotype reconstruction of unaffected individuals was performed using the program PHASE.

**Results:**

The recurrent *BRCA2 *mutation occurred in 1 of 60 (1.7%) women diagnosed with breast cancer before 41 years of age and one of 80 (1.3%) women with ovarian cancer. No mutation carriers were identified in the series of breast cancer cases diagnosed before age 80. Mutation carriers harboured one of two haplotypes, 7-3-9-3 – [3/4]-7, that varied with marker *D13S1701 *and which occurred at a frequency of 0.001. The genetic analysis of *D13S1695*, a polymorphic marker located approximately 0.3 cM distal to *D13S171*, did not favour a genetic recombination event to account for the differences in *D13S1701 *alleles within the haplotype. Although mutation carriers harbour genotypes that are frequent in the French Canadian population, neither mutation-associated haplotype was plausible in reconstructed haplotypes of 47 individuals of French Canadian descent.

**Conclusion:**

These results suggest that mutation carriers share a related ancestry; further supporting the concept that recurrent *BRCA1 *and *BRCA2 *mutations in the French Canadian population could be attributed to common founders. This finding provides further support for targeted screening of recurrent mutations in this population before large-scale mutation analyses are performed.

## Background

A significant proportion of breast and/or ovarian cancer families of French Canadian descent harbour specific recurrent mutations in *BRCA1 *and *BRCA2 *[1] which confer a significantly increased lifetime risk of developing young age of onset breast cancer and ovarian cancer [2-5]. The most common mutations were 4446C>T and 2953delGTAinsC in *BRCA1 *and, 6085G>T and 8765delAG in *BRCA2*. Haplotype analyses of polymorphic microsatellite repeat markers located within and flanking these cancer susceptibility genes have suggested that carriers of the same mutation share a common ancestry [1,6]. Previous analyses of French Canadian population have also shown that these recurrent *BRCA1 *and *BRCA2 *mutations accounted for about 13% (diagnosis before age 41 years) and 3% (diagnosis before 80 years of age) of breast cancer cases and 8% of ovarian cancer cases [7-9]. Recently, we reported a new *BRCA2 *mutation, 3398delAAAAG, found to occur in four of 169 unrelated breast and/or ovarian families of French Canadian descent [10]. A mutational screen consisting of the most common mutations, 4446C>T and 2953delGTAinsC in *BRCA1 *and, 6085G>T and 8765delAG in *BRCA2*, and 3398delAAAAG accounted for 62 of 74 (84%) mutation-positive French Canadian families with at least three cases of breast cancer (diagnosed before 66 years of age) and/or ovarian cancer. Based on these findings it was proposed that an initial screen for *BRCA *mutations in high risk families in this population include the new recurrent *BRCA2 *mutation [10].

To further characterize the contribution of this recently identified recurrent mutation in the French Canadian population, we estimated its frequency in breast and ovarian cancer cases unselected for family history of cancer. Given the precedence of the founder effect in this population, we also determined if carriers of the 3398delAAAAG mutation harbor a common haplotype that would suggest a shared ancestry.

## Methods

### Study population and mutation analyses

Three independently ascertained series of breast cancer and ovarian cancer cases unselected for family history of cancer were used to estimate the frequency of the *BRCA2 *mutation 3398delAAAAG. The breast cancer cases included a series of women diagnosed with cancer before 41 years of age (n = 60), and an independently ascertained series of women diagnosed with breast cancer before 80 years of age (n = 127). The ovarian cancer cases include a series of women (n = 80) diagnosed with invasive epithelial ovarian cancers ascertained with no age restriction. All women reported French Canadian ancestry and the cases have been described elsewhere [7-9]. The analysis for the presence of the *BRCA2 *3398delAAAAG mutation was performed on DNA extracted from peripheral blood lymphocytes of the breast or ovarian cancer cases as described previously [10].

Eleven index cases harbouring the 3398delAAAAG mutation affected with breast and/or ovarian cancer (Table 1) and, where possible, members from their families were genotyped for markers flanking *BRCA2*. Four of the eleven families (636, 762, 859 and 937) were reported previously [10]. The remaining seven families (1429, 1430, 1441, 1448, 1450, 1454, and 1458) were obtained from the Hereditary Cancer Clinics of McGill University and the Breast Clinic of the Division de gynécologie oncologique, Notre-Dame Hospital in Montreal. These two clinics represent the major genetic testing and counselling centres in the Province of Quebec and the only referring centres for Montreal and nearby regions. To our knowledge this study contains all known cases/families that harbour the *BRCA2 *3398delAAAAG mutation. The cancer site (breast or ovarian cancer) and age of diagnosis of the index case for genotype analysis in each family and those of relatives within three-degree relationships of the index case are shown in Table 1. Additional family members for phasing haplotypes were not available for families 1429 and 1430. The families harbouring the same mutation are not related to one another based on similarity of family structure and family names, and all index cases reported French Canadian ancestry.

**Table 1:** Family history of cancer in 3398delAAAAG *BRCA2 *mutation carriers

Family Number	Cancer site(s) and age of diagnosis of index case	Family history of breast and ovarian cancers and age at diagnosis of affected relatives	Family history of cancer at atypical sites and age at diagnosis of affected relatives
636	Br48	Br69;Ov69(M), Br30(MA)	CLL46(B), Thy;Lu50(MU), PSU50(MU), Pan44(MC)
762	Ov48;Ut48	BilBr55-57(S), Br65(PA), Br62(PC), Br45(PC), Br40(PC)	Lar60(PC), Hod/Lym55(PC), BD46(PC)
859	Br27	Br37(S), Br50(PA)	Pro55(PU), PSU63(PU)
937	Br33	Br47(S), Br44(PC), Br45(PC), Br39(PC), Br53(PC)	PSU61(F), Lu50(S),
1429	Br54	Br40;Bone46(S), Br44(MA), Br50(MA), Br52(MGM)	Pan66(M), PSU44(N), Co59(MU), Co(MU)
1430	Ov68;Pel72	Br67(S),	Leu76(S)
1441	Br52	Br49(MA), Br69(MA), Br68(MA), Ov50(MA), Br50(MA), Br58(MGM)	Lu64(MU), Brn41(MC)
1448	Ov76	Ov71(S), Br50(S), Br46(N)	
1450	Br45;Lu80	Br35(N)	Cx25(GD), Bone;Leu14(N), PSU47(N)
1454	Br26;Co44	Br45(PA), Br52(MA), Br34(MC)	Pro54(F), Mouth80(M), Lu62(PU), Pro73(PU), St58(PGF)
1458	Br45;FT45	Br52(S), Br52(PC), Br55(MC), Br53(MC)	Pro74(PU), Co43(PC), Co54(PC), Co54(PC), PSU60(PGF), Co40(MC), Cx43(MC)

Cancer site or type followed by age of diagnosis (if known): Breast (Br), bilateral breast (BilBr), ovarian (Ov), Uterus (Ut), prostate (Pro), colon (Co), chronic lymphoid leukemia (CLL), Leukemia (Leu), Lung (Lu), Pancreas (Pan), Brain (Brn), Larynx (Lar), Hodgkin's lymphoma (Hod/Lym), Bile duct (BD), Pelvic (Pel), Intestinal (In), Stomach (St), Cervix (Cx), Fallopian Tube (FT) and Primary site unknown (PSU). Affected relatives in parentheses: sister (S), brother (B), mother (M), father (F), daughter (D), granddaughter (GD), paternal aunt (PA), paternal uncle (PU), paternal cousin (PC), maternal aunt (MA), maternal uncle (MU), maternal cousin (MC), maternal grandmother (MGM), paternal grandfather (PGF) and niece (N). Multiple cancers in an individual are denoted by semicolon.

### Genotyping assays and haplotyping analysis

Genotype analysis was performed using seven polymorphic microsatellite repeat markers flanking *BRCA2*: *D13S260*, *D13S1699*, *D13S1698*, *D13S1697*, *D13S1701*, *D13S171*, and *D13S1695*. The order of the markers [11] and distances relative to each other and *BRCA2 *[12] are shown in Table 2. Genomic DNA from peripheral blood lymphocytes from index cases and family members were genotyped essentially as described previously [1,6]. Briefly, the PCR assays were performed in 12.5 ul volumes containing 50 ng genomic DNA; 1 × PCR buffer (Invitrogen); 200 uM of each dCTP, dGTP and dTTP; 10 uM dATP; 50 pmol of each primer [1]; 1.25 uCi ^35^S-dATP; and 0.5 U Taq DNA polymerase (Invitrogen). The reactions were amplified in a Perkin-Elmer 9600 Thermal Cycler for a total of 35 cycles at 95°C for 15 s, annealing at 55°C for 15 s, and 72°C for 30 s. The PCR products were diluted 10-fold with loading buffer (90% formamide, 10 mM EDTA pH8.0, 0.05% bromophenol blue, 0.05% xylene cyanol), denatured at 95°C for 5 min, and then 4 ul was loaded on a 5% acrylamide gel. The samples were electrophoresed at 70 W constant power at room temperature, transferred to Whatman paper, dried at 80°C on a vacuum gel drier, and then autoradiographed (Kodak) for 48–72 hours. The disease allele-associated haplotype was deduced by inspection of segregating genotypes in the families. The frequency of alleles was estimated by genotyping the 11 index cases representing an affected individual from each independently ascertained family harbouring the mutation and at least 47 unrelated, non-mutation carrier participants of French Canadian descent that were not affected with cancer.

**Table 2:** Genotypes of an index case from 3398delAAAAG mutation carrier families.

Family Number	Index case (mutation carrier)	Alleles						
		
		*D13S260*	*D13S1699*	*D13S1698*	*D13S1697*	*D13S1701*	*D13S171*	D13S1695
636* ^t^	N2254	**7 **9	**3 **1	**9 **6	**3 **2	**4 **4	**7 **9	**5 **3
762* ^t^	S359	**7 **7	**3 **4	**9 **9	**3 **2	5 **3**	**7 **2	**5 **5
859* ^t^	N2865	**7**,4	**3**,6	**9**,8	**3**,1	**4**,6	**7**,2	**5**,4
937* ^t^	S386-2	**7 **7	**3**,4	**9**,11	**3**,2	**4**,6	**7**,2	**5 **5
1450 ^t^	S1376	**7 **5	**3 **4	**9**,11	**3**,2	**4**,2	**7 **2	**5 **3
1448 ^t^	S1054	**7**,3	**3 **4	**9 **9	**3 **2	**4 **3	**7**,2	**5 **4
1429	N5445	**7**,4	**3 **3	**9**,11	**3**,2	**4**,**3**	**7 **7	**5**,2
1430	N5446	**7 **7	**3**,4	**9 **9	**3**,2	2,**3**	**7**,5	**5 **5
1441 ^t^	S1908	**7 **7	**3 **4	**9 **9	**3**,2	**4 **3	**7 **2	**5 **9
1454 ^t^	S922	**7 **8	**3 **3	**9 **3	**3 **3	**4 **4	**7 **9	**5 **5
1458 ^t^	S2160	**7 **7	**3 **4	**9 **3	**3 **2	**4 **2	**7 **9	**5 **3

Mutation carriers		Deduced haplotype						

3398delAAAAG		7	3	9	3	3,4	7	5
8765delAG		4	3	11	2	4	9	-

Families reported previously [10] (*). The 3398delAAAAG associated alleles are in boldface type, haplotypes not segregating with the *BRCA2 *mutant alleles are in italics, and genotypes of unphased alleles are separated by a comma. Phase was established by comparing genotypes of two to nine family members per family where additional members were available for analysis^t^. Alleles common in the French Canadian population reported previously [6]. The physical distance between *D13S260 *to *D13S1695 *is about 3.5 Mb [15]; *BRCA2 *is located between *D13S1697 *and *D13S701 *[11] The intermarker distances are *D13S260 *– 1.0 cM – *D13S1699 *– 0.7 cM – *D13S1698 *– 0.6 cM – *D13S1697 *– 0.2 cM – *BRCA2 *– 0.5 cM – *D13S1701 *– 0.6 cM – *D13S171 *– 0.3 cM – *D13S1695 *[12].

### Haplotype reconstruction

The genotypes of 47 French Canadians unaffected with cancer were used to reconstruct haplotypes that were not associated with *BRCA2 *mutation carriers. Haplotypes were reconstructed using the program PHASE version 2.1 [13,14] based on genotypes deduced from the analysis of the six microsatellite markers: *D13S260*, *D13S1699*, *D13S1698*, *D13S1697*, *D13S1701*, and *D13S171*. The physical distances of the genetic markers required for haplotype reconstruction were derived from the Human Genome Browser assembly hg17 (May 2004 version) from the UCSC Genome Bioinformatics [15].

## Results and discussion

Haplotype analysis was initiated with six polymorphic markers, *D13S260*, *D13S1699*, *D13S1698*, *D13S1697*, *D13S1701*, and *D13S171 *spanning a 3.6 cM interval containing *BRCA2 *(Table 2). Haplotypes were phased for all markers for only five of the eleven families (636, 762, 1441, 1454 and 1458) due to limitations of reagents from family members or inability to phase all genotypes based on non-informativity of markers (Table 2). Based on the results from these families, two haplotypes were observed, 7-3-9-3 – [3/4]-7, which varied for the *D13S1701 *alleles. All mutation carriers of unphased genotypes harboured alleles consistent with either haplotype. The disease associated alleles in 3398delAAAAG carriers were distinct for markers *D13S260*, *D13S1698*, *D13S169*, and *D13S171 *when compared with those found to segregate with carriers of the 8765delAG mutation [6], the most common *BRCA2 *mutation reported in French Canadian cancer families (Table 2). The most common allele(s) in unaffected individuals for four of the six markers tested were those associated with 3398delAAAAG (Table 3). Although the alleles associated with the recurrent mutation are frequent in the population (Table 3), neither mutation-associated haplotype was plausible in reconstructed haplotypes of 47 French Canadians unaffected with cancer. Furthermore, the *D13S1697 *disease segregating allele, situated 0.2 cM immediately proximal to *BRCA2*, was not the most common allele in the unaffected French Canadians. Under the assumption of linkage disequilibrium, the frequency of either haplotype associated with the 3398delAAAAG mutation was estimated at 0.001. This result suggest that the 3398delAAAAG mutation is not carried on haplotype comprised of the most common alleles in the French Canadian population and provides support for the notion that the haplotypes of the mutation carriers are identical by descent in the French Canadian population.

**Table 3:** Allele frequencies in non-carriers or French Canadians unaffected with cancer and carriers of 3398delAAAAG mutation.

Allele	Marker (number of subjects)											
	*D13S260*		*D13S1699*		*D13S1698*		*D13S1697*		*D13S1701*		*D13S171*	
	*NC *(45)	C (11)	*NC *(43)	C (11)	*NC *(51)	C (11)	*NC *(35)	C (11)	*NC *(41)	C (11)	*NC *(41)	C (11)
0	-	-	-	-	-	-	-	-	-	-	-	-
1	-	-	0.05	0.05	-	-	0.01	0.05	0.01	-	0.02	-
2	0.02	-	0.02	-	0.02	-	***0.74***	0.41	0.07	0.14	***0.40***	0.27
3	0.19	0.05	***0.62***	***0.59***	0.05	0.09	0.24	***0.55***	0.22	0.23	0.02	-
4	0.08	0.09	0.27	0.32	0.03	-	-	-	***0.23***	***0.50***	-	-
5	0.09	0.05	0.02	-	0.02	-	-	-	0.18	0.05	0.01	0.05
6	0.06	-	0.01	0.05	0.08	0.05	-	-	0.21	0.09	-	-
7	***0.32***	***0.73***	-	-	0.04	-	-	-	0.06	-	0.33	***0.55***
8	0.11	0.05	-	-	0.02	0.05	-	-	-	-	-	-
9	0.12	0.05	-	-	***0.40***	***0.68***	-	-	0.01	-	0.21	0.14
10	0.01	-	-	-	0.04	-	-	-	-	-	-	-
11	-	-	0.01	-	0.30	0.14	-	-	-	-	-	-
12	-	-	-	-	-	-	-	-	-	-	-	-
13	-	-	-	-	-	-	-	-	-	-	-	-

The figures in bold type show the most common allele. Alleles in italics associated with 3398delAAAAG mutation. NC and C refer to non-carriers and carriers of this mutation, respectively.

In order to establish if a recombination event could account for the differences in the haplotypes, we genotyped an additional polymorphic microsatellite repeat marker, *D13S1695*, which is situated approximately 1.4 cM from the 5' end of *BRCA2 *[11,12]. Genotypes of phased alleles flanking *BRCA2 *in mutation carriers would not favour a genetic recombination event. The variance in allele size of one tetranucleotide repeat unit within the *D13S1701 *allele, may have been due to a mutation caused by slippage during DNA replication of this repeat sequence. While the frequency of DNA replication errors in repeat sequences and the affect of repeat unit size are not known, studies have shown a positive correlation between repeat length and mutation rates [16]. Without further genetic analysis it was not possible to determine if *D13S1701 *exhibits a higher mutation frequency than other markers in the *BRCA2 *region or that the mutation occurred early in the development of the French Canadian population. The later possibility is suggested by the observation that both haplotypes could be associated with 3398delAAAAG in more than one unrelated mutation carrier.

The 3398delAAAAG mutation has been previously reported eight times in the Breast Cancer Information Core Database [17], where all carriers were either of Western European or French Canadian descent [18]. Presently it is not known if the French Canadian carriers reported in this database are those identified in our hereditary cancer clinics and thus related to members of the families in the present study. The mutation was identified in 1 of 60 (1.7%) women with breast cancer diagnosed before 41 years of age and none in the independently ascertained series of 127 women with breast cancer diagnosed before 80 years of age, and 1 of 80 (1.3%) women with ovarian cancer. The frequency of mutation carriers in these series is keeping with the observation that four of 169 (2.4%) high-risk breast and/or ovarian cancer families of French Canadian descent harboured this mutation in that this recurrent mutation is not as common as the 4446C>T in *BRCA1 *and 6085G>T and 8765delAG in *BRCA2 *in this population [7-10]. The mutation-positive carriers were represented by families 636 and 859 (Table 2) and thus mutation-positive carriers in these families harbour genotypes in common with other carriers of this mutation. Carriers of this mutation in our series of eleven families appear not to share a recent relationship based on inspection of pedigrees, although all families cite the Montreal region and Central Quebec as place of origin of grandparents, unlike carriers of either the 8765delAG or 4446C>T *BRCA *mutations which reported grandparental origins in various regions of Quebec [1]. Thus it would be interesting to compare haplotypes of all carriers of 3398delAAAAG mutation to determine those in the French Canadian population share common ancestry with Western Europeans.

The 3398delAAAAG mutation is located within the Ovarian Cancer Cluster Region (OCCR) in exon 11 of *BRCA2*, which has been associated with increased risk for ovarian cancer in breast cancer families [19]. Although the presence of at least one ovarian cancer in high risk French Canadian families is a strong predictor of a *BRCA1 *mutation [1], four of the nine mutation carrier high risk families (families 636, 762, 1441, and 1448) in the present study contained at least one case of ovarian cancer (Table 1). One of these families, number 1448, also had two cases of ovarian cancer. The index case for testing in family 1430 also had ovarian cancer, although this family would not have been selected for mutation analysis based on our inclusion criteria for mutation screening [1,10]. As observed in our previous studies of French Canadian cancer families [10], with one exception in family 762, the age of diagnosis of ovarian cancer was higher than the mean age of diagnosis of ovarian cancer (age 56 years) in the Canadian population [20]. This is particularly evident in family 1448 where both cases of ovarian cancer were diagnosed after age 70 years (Table 1). The index case in Family 1458 diagnosed with breast cancer at age 45 years was also diagnosed with a primary cancer of the fallopian tube, (Table 1), a type of gynecological cancer which shares many clinicopathologic features in common with epithelial ovarian cancers. Germline BRCA mutations have been reported in women with fallopian tube cancers supporting the notion that these cancers share a common molecular pathogenesis with ovarian cancers [21]. Taken together, five of the nine high risk families contain cases of either ovarian or fallopian tube cancers. Although the association with mutations in the OCCR region and ovarian cancer risk in the French Canadian high-risk population is not known, the proportion of high-risk 3398delAAAAG mutation-positive families with at least one case of ovarian cancer is not significant different from those harbouring the 6503delTT mutation (20% (2/10)) located within the OCCR region and the 8765delAG mutation (29% (5/17)) located outside of the OCCR region, the other two recurrent *BRCA2 *mutations in the French Canadian population [10].

## Conclusion

This study provides further evidence towards common origins of the *BRCA *mutations in the French Canadian population of Quebec and further rationalizes a targeted screening of recurrent mutations in this population before large-scale mutation analyses are performed.

## Competing interests

The author(s) declare that they have no competing interests.

## Authors' contributions

KKO participated in study design, performed data analysis and drafted the manuscript. GL, SLA, and ZS performed genotyping assays. CP, WDF, PG and DP recruited the participants and collected clinical information from participants. M-M M-M, DP and PNT were responsible for coordinating DNA banks from participants. PNT designed and coordinated the study and contributed in drafting the manuscript. All authors have read and approved the final manuscript.

## Pre-publication history

The pre-publication history for this paper can be accessed here:


